# Generation of spin waves by a train of fs-laser pulses: a novel approach for tuning magnon wavelength

**DOI:** 10.1038/s41598-017-05742-x

**Published:** 2017-07-18

**Authors:** I. V. Savochkin, M. Jäckl, V. I. Belotelov, I. A. Akimov, M. A. Kozhaev, D. A. Sylgacheva, A. I. Chernov, A. N. Shaposhnikov, A. R. Prokopov, V. N. Berzhansky, D. R. Yakovlev, A. K. Zvezdin, M. Bayer

**Affiliations:** 1 Lomonosov Moscow State University, 119991 Moscow, Russia; 2 Russian Quantum Center, Skolkovo, 143025 Moscow, Russia; 3 Experimentelle Physik 2, TU Dortmund, D-44221 Dortmund, Germany; 4 Ioffe Institute, Russian Academy of Sciences, 194021 St. Petersburg, Russia; 5 Prokhorov General Physics Institute, Russian Academy of Sciences, 119991 Moscow, Russia; 6 Vernadsky Crimean Federal University, Vernadsky Ave. 4, 295007 Simferopol, Russia; 7 Faculty of Physics, National Research University Higher School of Economics, Myasnitskaya 20, Moscow, 101000 Russia

## Abstract

Currently spin waves are considered for computation and data processing as an alternative to charge currents. Generation of spin waves by ultrashort laser pulses provides several important advances with respect to conventional approaches using microwaves. In particular, focused laser spot works as a point source for spin waves and allows for directional control of spin waves and switching between their different types. For further progress in this direction it is important to manipulate with the spectrum of the optically generated spin waves. Here we tackle this problem by launching spin waves by a sequence of femtosecond laser pulses with pulse interval much shorter than the relaxation time of the magnetization oscillations. This leads to the cumulative phenomenon and allows us to generate magnons in a specific narrow range of wavenumbers. The wavelength of spin waves can be tuned from 15 μm to hundreds of microns by sweeping the external magnetic field by only 10 Oe or by slight variation of the pulse repetition rate. Our findings expand the capabilities of the optical spin pump-probe technique and provide a new method for the spin wave generation and control.

## Introduction

Nowadays moving from electric currents to spin waves (SW), or magnons, is considered very promising for the further development of telecommunication technologies. Thereby losses should be diminished and processing speed tremendously increased^1–3^. Spin excitations and their detection can be accomplished all-optically by the pump-probe technique^4^. The femtosecond (fs) laser pump pulses cause magnetization oscillations in a magnetic sample either by the ultrafast demagnetization, or by the magnetocrystalline anisotropy, or by the inverse Faraday effect^4–14^. The probe pulse hits the sample at some delay with respect to the pump pulse and provides information about the magnetization direction through its polarization change, using the Faraday or Kerr rotation magneto-optical effects.

Non-local spin precession, i.e. propagating SW excitation, was first optically excited by M. van Kampen *et al*.^15^ in ferromagnetic metals (permalloy, nickel) by local heating of the sample with 100-fs-laser pulses. Non-thermal optical excitation of the SWs was demonstrated by Satoh *et al*.^16^ in magnetic dielectrics. This breakthrough work revealed the advantages of the optical approach. In detail, the authors of ref. 16 used focused femtosecond laser pulses with circular polarization. The light pulses induced an effective magnetic field **H**_F_ inside the magnetic medium due to the inverse Faraday effect^17, 18^. This field exists in the medium during propagation of light through the sample and deflects the magnetization from its equilibrium orientation, thereby launching SWs. The appealing feature of this approach is that it allows one to originate a point source of SWs by focusing light in a micron- or even submicron-sized spot. Secondly, the optical approach is contactless and the excitation spot can be easily shifted across the sample from one point to another. Moreover, spatial shaping and sizing of the light pulse modify the spectrum and distribution of the SW wavenumbers^16^. In the ensuing papers^19–22^ additional advantages of this technique were identified, including phase control and switching between different types of SWs.

In all aforementioned works, SWs were generated in a “single pulse” regime when the laser pulses hit the sample relatively rare so that the magnetization oscillations excited by a previous pulse have already vanished before the next one. As a result, the spectrum of the SWs is rather broad. Double-pulse optical excitation was explored in refs 9 and 23. Only few papers have addressed the magnetization control in a multiple-pulse regime so far^24, 25^. In ref. 24 the magnetization was influenced by a sequence of picosecond acoustic pulses. Optical generation of SWs by a train of laser pulses was demonstrated in ref. 25, which allowed one to increase the SW amplitude significantly.

In the present work we identify a novel feature of the periodic optical excitation of SWs. In particular, we excite magnetization of the sample with a sequence of circularly polarized pulses at a high repetition rate so high that the interval between pulses is shorter than the decay time of oscillations, which provides a phenomenon of frequency selection: Those SWs whose frequencies are multiples of the laser pulse repetition rate are mostly supported while SWs with frequencies that are semi-integer multiples to the laser pulse repetition rate become suppressed. As a result, SWs are generated in a specific narrow range of wavenumbers. Furthermore, modifying the laser pulse repetition rate or the strength of the external magnetic field provides significant tunability of the SW wavelength. In our particular case, we have chosen a magnetic film with such magnetic parameters and thickness that it allows us to change the SW wavelength by about 20 times from 15 μm to 290 μm with just a tiny variation of the external magnetic field by a few percent.

## Experimental

The experimental studies were performed on two thin magnetic films of bismuth iron-garnet epitaxially grown on gadolinium gallium garnet substrates. The first film (sample-A) is 0.6-μm-thick, has composition of (Bi_1.4_Y_1.6_)(Al_1.6_Sc_0.2_Fe_3.2_)O_12_ and was grown on a (CaMgZrGd)_3_Ga_5_O_12_ substrate, while the other one (sample-B) is 5.0-μm-thick, has composition of (Bi_0.9_Lu_1.4_Tm_0.4_Y_0.2_Sm_0.1_)(Fe_4.7_Ga_0.3_)O_12_ and was grown on a Gd_3_Ga_5_O_12_ substrate^26, 27^. Due to the difference in composition the two samples differ in magnetic parameters: for the sample-A the magnetization of saturation is 4*πM*_s_ = 348 Oe, the uniaxial anisotropy constant is *K*_*u*_ = 10^3^ erg ∙ cm^−3^ and the cubic anisotropy constant is *K*_1_ = −5 ∙ 10^3^ erg ∙ cm^−3^ and for the sample-B 4*πM*_s_ = 1000 Oe, *K*_*u*_ = 3.0 ∙ 10^4^ erg ∙ cm^−3^ and *K*_1_ = −1.2 ∙ 10^3^ erg ∙ cm^−3^.

**Figure 1:**
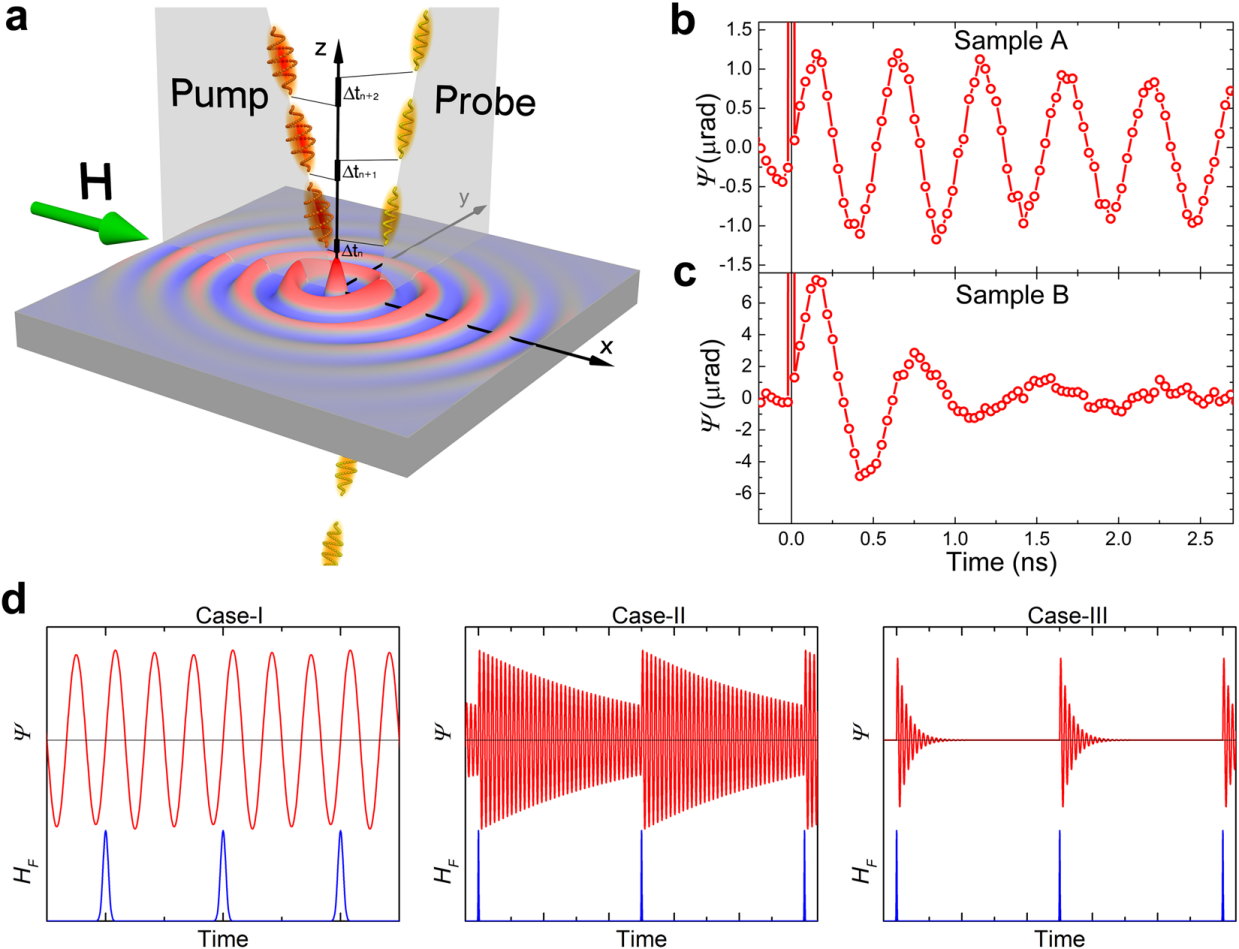
Excitation of the magnetization dynamics by the periodic pulses. **(a**) Scheme of the pump-probe experiment. The sample is illuminated with pairs of pump and probe pulses. The time delay between the pump and probe pulses varies Δ*t*_*n*_ from 0 to 2.6 ns. (**b**,**c)** Oscillations of the Faraday rotation of the probe beam polarization, demonstrating magnetization precession in the sample-A (**b**) and B (**c**) excited by the 80MHz-laser. The external magnetic field is 590 Oe. (**d)** Three considered cases of the ratio of the interval between pump pulses (*T*) and decay time of the magnetization precession (*τ*): $\frac{{T}_{1}}{{\tau }_{A}}\ll 1$ (case-I), $\frac{{T}_{2}}{{\tau }_{A}} \sim 1$ (case-II), and $\frac{{T}_{2}}{{\tau }_{B}}\gg 1$ (case-III). Red curve represents the magnetization precession and the blue one represents effective magnetic field of the inverse Faraday effect.

In the pump-probe technique, the magnetization dynamics is excited by circularly polarized pump pulses and is observed by the variation of the Faraday rotation angle *Ψ* of the linearly polarized probe pulses propagating through the sample at some time delay with respect to the pump pulses (Fig. 1a). Generation of the SWs is studied by spatial displacement of the pump beam with respect to the probe one. The external magnetic field up to 600 Oe is applied in the sample plane using an electromagnet. All measurements are performed at room temperature.

The experiments were performed using two pump-probe set-ups with lasers having different pulse repetition rates of 1 GHz and 80 MHz, corresponding to intervals between pulses of *T*_1_ = 1 ns and *T*_2_ = 12.5 ns, respectively. In both cases the pump and the probe beams are focused onto the sample using a single reflective microscope objective with a magnification factor of 15 comprising 4 sectors through which the light can enter the sample. The second objective in transmission geometry is used to collect and collimate the probe beam to the polarimetric detection scheme, which comprises a polarization bridge and a balanced photoreceiver. Pump and probe beams are incident at angles of 17 degrees in planes orthogonal to each other (the XZ- and YZ-planes in Fig. 1a). The pump and probe pulses are focused into spots with radii of *r*_0_ = 5 µm.

The first setup is based on asynchronous optical sampling (ASOPS)^28^. Two independent Ti:Sapphire laser oscillators (Gigajet TWIN 20c/20c) for the circularly polarized pump and the linearly polarized probe beams emit 50 fs pulses at a rate of about *f* = 1 GHz. The center wavelengths of the pump and the probe beam are tuned to 810 nm and 850 nm, respectively. The pump pulse energy is around 50 pJ while the probe pulse energy is about 3 pJ. The repetition frequencies of the oscillators are synchronized to each other with a small offset of Δ*f* = 20 kHz using a TL-1000-ASOPS unit. As a result, the relative time delay between pump and probe pulses is repetitively ramped from zero to 1 ns within a scan time of 50 µs. In this case the ultrafast signal is linearly stretched in time by a factor about $\frac{f}{{\rm{\Delta }}f} \sim 5\times {10}^{5}$ and made accessible to fast data acquisition electronics. Each of the transient traces is accumulated 10^6^ times corresponding to an accumulation time of about 50 s. The angle of Faraday rotation for the probe beam polarization *Ψ* is measured using a polarization bridge which comprises a Wollaston prism and a 10-MHz balanced photodetector. The amplified signal is sent to a high speed multi-channel digitizer triggered by the ASOPS system at the frequency of 2 kHz. The resulting Faraday rotation angle is given by *Ψ*(*t*) = *dU*(*t*)/4*U*_*dc*_, where *dU*(*t*) is the time-resolved differential signal and *U*_*dc*_ is the average intensity measured with one of the photodiodes.

The second setup comprises a Spectra Physics Mai Tai HP Ti:Sapphire laser, operating at 80.68 MHz repetition rate, which is combined with a Spectra-Physics Inspire Auto 100 optical parametric oscillator. The duration of laser pulses is 200 fs. The delay line is formed by a retroreflector mounted on a linear stage. The time delay between pump and probe pulses is varied from −0.5 to 2.6 ns, where zero time delay corresponds to simultaneous propagation of the pump and probe pulses through the sample. The pump and probe beam radii *r*_0_ = 5 µm. Energy of the pump pulse is 370 pJ, wavelength is 620 nm and energy of the probe pulse is around 10 pJ, wavelength is 820 nm. The polarization of the pump pulses was modulated at 40 kHz frequency between right- and left-handed (σ+ and σ−) circular by a photo elastic modulator. A balanced diode detector in combination with a lock-in amplifier was set to the modulation frequency to measure the linearly polarized probe signal after propagation through the sample.

## Results

The laser with 80 MHz repetition rate excites in the sample-A magnetization precession with slightly decaying amplitude as is seen by probing the area illuminated by the pump pulses (Fig. 1b). The decay time is *τ*_*A*_ = 13 ns, so that $\frac{{T}_{2}}{{\tau }_{A}} \sim 1$. Considerable oscillations excited by the previous pulse are still visible at negative time delay. For sample-B the decay time is significantly shorter: *τ*_*B*_ = 0.8 ns, and $\frac{{T}_{2}}{{\tau }_{B}}\gg 1$ (Fig. 1c). For the 1GHz-laser the pulse interval is much shorter and, therefore, $\frac{{T}_{1}}{{\tau }_{A}}\ll 1$ and $\frac{{T}_{1}}{{\tau }_{B}} \sim 1$. Therefore, we have three different situations here: case-I, where the oscillation decay occurs much slower compared to the pulse interval $(\frac{{T}_{1}}{{\tau }_{A}}\ll 1)$; case-II, where the oscillation decay time is comparable to the pulse interval $(\frac{{T}_{2}}{{\tau }_{A}} \sim 1)$; and case-III, where the oscillations decay is much faster in comparison to the pulse interval $(\frac{{T}_{2}}{{\tau }_{B}}\gg 1)$. One therefore expects that the spectra of the generated SWs differ in these cases. The immediately emerging question is whether it is possible to use appropriate excitation conditions to modify the SW spectrum significantly with respect to that in the single-pulse excitation regime in order to generate SWs with a much narrower spectrum of wavenumbers that can be tuned by a magnetic field?

**Figure 2:**
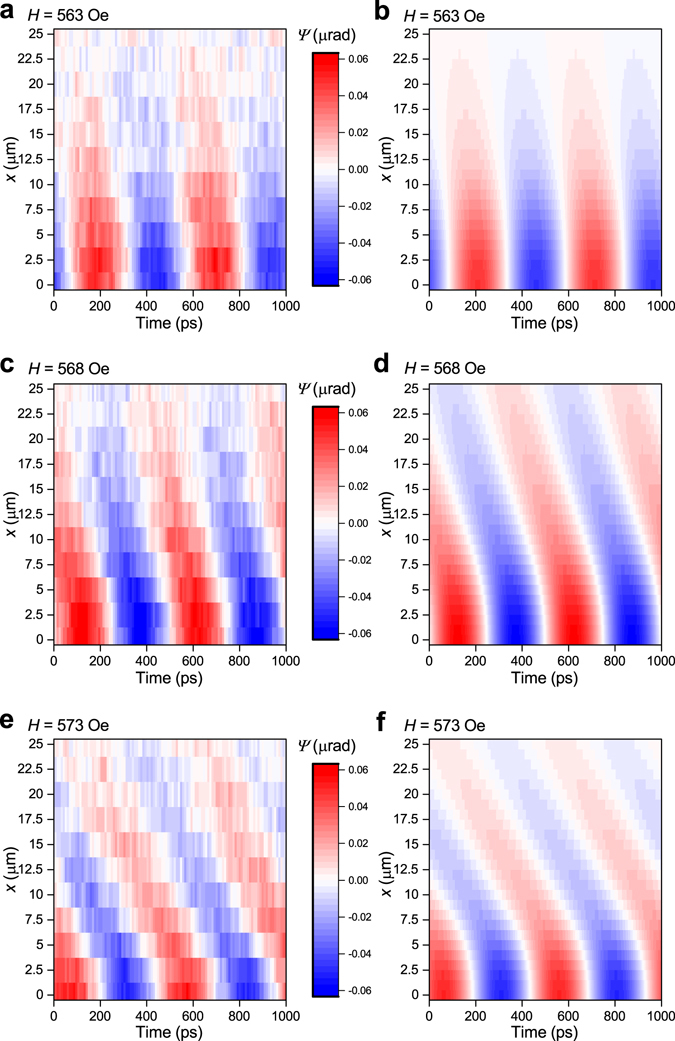
Influence of the external magnetic field on the propagation of SWs excited by the 1GHz-laser in sample-A (case-I). Color contour plots of experimentally measured (**a,c,e**) and calculated (**b,d,f**) oscillations of the out-of-plane magnetization component in time and space, detected through the Faraday rotation *Ψ*(*x*, *t*) at three different values of the external magnetic field *H* = 563 Oe (**a,b**), 568 Oe (**c,d**), and 573 Oe (**e,f**). For the calculations we use the Gilbert damping constant *α* = 4 × 10^−3^ which provides the best correspondence with the experimental data.

Let us first consider in detail the case-I where the pump pulses impinge on the sample with an interval much shorter than the decay time of the excited magnetic oscillations and therefore a cumulative effect of the laser pumping is expected. If the probe beam is shifted away from the excitation spot the oscillations are still observed but their amplitude and phase change as shown by the red-blue colored contour plots of the Faraday rotation angle at different delay times (*t*) and displacements (*x*) (Fig. 2a,c,e). The propagation of the spin waves is clearly seen as becomes evident for shifts larger than 4 μm when the overlap between the pump and probe spots becomes negligible. The decrease of the oscillation amplitude with increasing distance is related to the spread of SW energy, while the linear change of the SW phase is due to propagation of the SW at a given phase velocity. The experimental data are well reproduced by calculations based on the SW dispersion in sample-A (Methods) (Fig. 2b,d,f).

**Figure 3:**
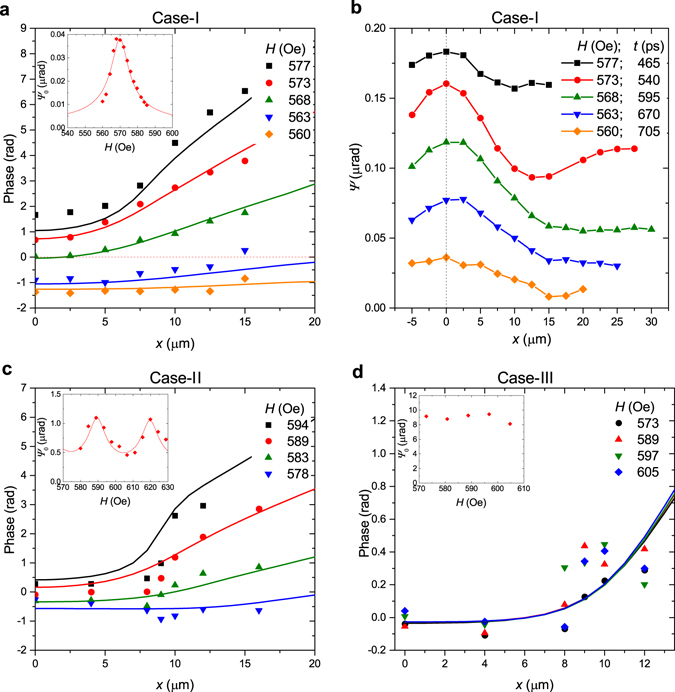
Influence of the external magnetic field on the phase and wavenumber of the spin waves optically generated by the 1GHz-laser in sample-A (**a,b**), and by the 80MHz-laser in sample-A (**c**), and sample-B (**d**). The spin waves are traced along the *x*-axis. (**b**) Distribution of the SW magnetization component parallel to the probe beam (measured by Faraday rotation, *Ψ*) at the moments when it is maximal at *x* = 0. Insets: amplitude of the spin waves (measured by Faraday rotation, *Ψ*_0_) at *x* = 0 versus external magnetic field. Experimental data are shown as dots while calculation results are presented by solid curves. For the calculations of the phase *ξ*(*x*), *α* = 4 × 10^−3^ for the sample-A (**a–c**) and *α* = 20 × 10^−3^ for the sample-B (**d**) are taken. For the calculations of the SW amplitude, *α*_*SW*_ = 6.5 × 10^−3^ (insets in **a** and **c**).

The experimental data can be described as *Ψ*(*x*, *t*) = *Ψ*_0_(*x*)e^−*t*/*τ*^sin(2*πνt* + *ξ*(*x*)), where *Ψ*_0_(*x*), *ν* and *ξ*(*x*) are the oscillation amplitude, frequency and phase, respectively, and *τ* is the decay time. Remarkably, *ξ*(*x*) and *Ψ*_0_(*x*) depend strongly on magnetic field (Fig. 3a, and inset). At the same time, the SW frequency *ν* = 2 GHz remains nearly the same, which is an integer multiple of the 1GHz-laser pulse repetition rate. This is a clear hint that periodicity of the optical excitation is a key factor in the observed phenomena.

Amplitude of the generated SWs has pronounced maximum at *H* = 570 Oe (inset of Fig. 3a). Slight deviation of *H* by 10 Oe makes the signal hardly observable and *Ψ*_0_ drops by 5 times. Going further from the resonant magnetic field decreases the signal amplitude below the noise level.

Starting from *x* = 4 µm the function *ξ*(*x*) becomes linear (Fig. 3a), *ξ*(*x*) can be represented as *ξ*(*x*) = *k*_*m*_ *x*, which indicates generation of SWs with mean wavenumber *k*_*m*_. For the external magnetic field varying from *H* = 560 Oe to 577 Oe the slope of the linear part of *ξ*(*x*) increases tremendously by about 20 times which corresponds to growth of *k*_*m*_ from 0.022 rad μm^−1^ to 0.4 rad μm^−1^. At the same time, the mean wavelength of SWs, *λ*_*m*_ = 2*π*/*k*_*m*_, varies from 15 to 290 μm. The change of SW wavelength with external magnetic field is directly visible in the plots demonstrating *Ψ*(*x*, *t* = *const*) measured at fixed moments (Fig. 3b).

In the case-II (SW excitation in sample-A with the 80MHz-laser) the periodicity of pulses remains important for the observed spin dynamics. Even in spite of the fact that the oscillation amplitude is reduced by a factor of 3 at the moment of subsequent pulse arrival (since *T*_2_ ~ *τ*_*A*_ ~ 13 ns), the dependency of *ξ*(*x*) varies with external magnetic field noticeably (Fig. 3с). Consequently, in this regime the cumulative effect of the pump pulses is still present.

Finally, we consider the case-III, when the oscillations have decayed significantly before the next pulse arrival. In contrast to the previous data, the rate of the phase variation and, therefore *k*_*m*_, do not change with magnetic field within the accuracy of the experiment (Fig. 3d). The SW amplitude remains almost constant as well. Therefore, for $\frac{{T}_{2}}{{\tau }_{B}}\gg 1$ (*τ*_*B*_ = 0.8 ns) the cumulative effect is absent and no tuning of the SW wavelength is possible.

Cases-I and II differ from case-III also in terms of the SW amplitude variation with magnetic field (insets in Fig. 3a, c and d): while for the first two cases *Ψ*_0_ depends strongly on *H* (insets in Fig. 3a,c), for the third one *Ψ*_0_ remains almost constant (insets in Fig. 3d). In the cases-I and II, *Ψ*_0_(*H*) has maxima at the *H*-strengths when the oscillation frequency *ν* is a multiple of the laser repetition rate. Such behavior points toward the phenomenon of oscillation synchronization. It is important to clarify how the synchronization efficiency depends on *ν*.

## Discussion of the observed phenomena

### Theoretical model for the uniform magnetization precession under periodic pumping

In order to estimate the efficiency of the optical excitation of SWs with different wave vectors and to explain the observed results we will consider the magnetization precession within the illuminated spot of the film. Let us consider uniform precession of the magnetization **M**(*t*) under the influence of the temporally periodic effective magnetic field of the inverse Faraday effect, **H**_*F*_(*t*). The Lagrange function, *L*(*t*), and the dissipation Rayleigh function, *R*(*t*), in spherical coordinates (Fig. 1a) can be written as:

1a $$L=\frac{M}{\gamma }(1-\cos \,\theta )\dot{\phi }-U,$$

1b $$R=\alpha \frac{M}{2\gamma }({\dot{\theta }}^{2}+{\sin }^{2}\theta {\dot{\phi }}^{2}),$$

where *γ* is the gyromagnetic ratio, *α* is the Gilbert damping constant, *θ* and *φ* are the polar and azimuth angles of **M** in the spherical coordinate system with the z-axis along the film normal (Fig. 1a) and the free energy density *U* of the magnetic film *U* = −*K* sin^2^ *θ* − *MH* sin *θ* cos *φ* − *MH*_*F*_(*t*) cos *θ*.

When the film is magnetized in-plane it is convenient to change to the angle *θ*_1_ = π/2 − *θ*, since *θ* ≪ 1, which allows one to linearize Eq. (1). In the case of small Gilbert damping (*α* ≪ 1) Eq. (1) lead to the following Euler equations for the magnetization dynamics:

2a $${\dot{\theta }}_{1}={\omega }_{H}\phi -\alpha ({\omega }_{a}+{\omega }_{H}){\theta }_{1}+\alpha {\omega }_{F},$$

2b $$\dot{\phi }=-({\omega }_{a}+{\omega }_{H}){\theta }_{1}+{\omega }_{F}-\alpha {\omega }_{H}\phi ,$$

where *ω*_*H*_ = *γH*, *ω*_*a*_ = *γ*(4*πM* − 2*K*_*u*_/*M*), *ω*_*F*_ = *γH*_*F*_(*t*). Note that *θ*_1_ is directly related to the observed Faraday angle: *θ*_1_ = *Ψλn*/*πgl*, where *n* and *g* are the refractive angle and magnetooptical gyration of the magnetic film and *l* is its thickness^29^.

If the pulse duration Δ*t* is sufficiently small (Δ*t* ≪ *T*, ${\,{\rm{\omega }}}_{H}^{-1}$) and the pulses arrive periodically, then the field of the inverse Faraday effect can be represented as a series of *δ*-functions of amplitude *h*: ${H}_{F}(t)=h{\rm{\Delta }}t\sum _{m=0}^{+\infty }\delta (t-mT)$.

The solution of Eq. (2), *θ*_1_(*t*), is a periodic function with period ${\theta }_{1}(t)={\theta }_{0}\,\sin (2{\rm{\pi }}{\rm{\nu }}t+\xi ){e}^{-t/{\tau }_{0}}$ at *mT* < *t* < (*m* + 1)*T*, *m* ≫ 1. Here $\nu ={\nu }_{0}\sqrt{1-{({\nu }_{0}{\tau }_{0})}^{-1}}\approx {\nu }_{0}$, ${\nu }_{0}=\frac{1}{2\pi }\sqrt{{\omega }_{H}({\omega }_{H}+{\omega }_{a})}$, and *τ*_0_ = 2/*α*(2*ω*_*H*_ + *ω*_*a*_). Amplitude, *θ*_0_, and phase, *ξ*, are given by:

3a $${\theta }_{0}=\frac{{\gamma }^{2}Hh{\rm{\Delta }}t}{2\pi \nu }{(1-2{e}^{-T/{\tau }_{0}}\cos 2\pi \nu T+{e}^{-2T/{\tau }_{0}})}^{-\frac{1}{2}}$$

3b $$\xi ={\rm{atan}}[{e}^{-T/{\tau }_{0}}\,\sin \,2\pi \nu T(1-{e}^{-T/{\tau }_{0}}\,\cos \,2\pi \nu T)]$$

### Frequency dependence of the amplitude and phase of magnetization precession in the illuminated magnetic film area

Even though the theoretical model above is derived for uniform oscillations in space, it can be applied for calculation of the oscillation amplitude within the pump spot when SWs are generated. The SWs spread the oscillation energy away from the area illuminated by the pump pulses. Moreover, the broad spectrum of SWs also causes a decrease of the observed oscillation amplitude due to desynchronization and destructive interference of different harmonic modes. These phenomena lead to excess damping of the oscillations in the pumped area. Therefore, generation of SWs can be taken into account by introducing an effective damping constant *α*_*SW*_ that is larger than the Gilbert one in Eqs. (1–3). The constant *α*_*SW*_ can be found from fitting the experimentally measured dependence of the oscillation amplitude on magnetic field, *Ψ*_0_(*H*), with Eq. (3) taking into account that *θ*_1_ ~ *Ψ* (solid curves in the insets of Fig. 3a,c). Thus, we get for the cases-I and II *α*_*SW*_ = 6.5 × 10^−3^.

In accordance with Eq. (3a), for periodic pumping, the SW amplitude depends on the SW frequency. If the frequency is equal to or a multiple of the laser pulse repetition rate then synchronization between the magnetization precession and the pump pulses takes place and the SW amplitude increases. On the contrary, for the SWs with frequencies that are semi-integer multiples of the laser pulse repetition rate the SW amplitude becomes suppressed. The phase of the SWs also varies with changing the SW frequency (see Eq. (3b)). In particular, in the synchronization regime the phase vanishes which allows perfect matching of the magnetization oscillations excited by consecutive pump pulses. As the SW frequency is determined by the external magnetic field the SW amplitude and phase can be tuned by the magnetic field.

The dependence of *θ*_0_(*ν*) is mostly determined by the effective field of the inverse Faraday effect, *h*, and the ratio *T*/*τ*, where *τ* = 2/*α*_*SW*_(2*ω*_*H*_ + *ω*_*a*_). In fact, *θ*_0_(*ν*) determines efficiency of the oscillation synchronization.

For relatively frequent pump pulses such that *T* ≪ *τ*_0_, which corresponds to the excitation of sample-A with the 1GHz-laser, the pumping leads to a cumulative effect. As a result, *θ*_0_(*ν*) has sharp peaks with maxima at *v* = *m*/*T*. At these frequencies synchronization of the magnetization oscillations excited by consecutive laser pulses occurs. It explains observed significant growth of the SW amplitude (inset in Fig. 3a). Near the maxima, *θ*_0_(*ν*)can be approximated by a Lorentzian with full width at half maximum (FWHM) *δν* = 1/*πτ*_0_. Because *T* ≪ *τ*, we have $\delta \nu \ll \frac{1}{T}$.

When *T*/*τ* ≥ 1, corresponding to excitation of both samples with the 80MHz-laser, *θ*_0_(*ν*)is given by a harmonic oscillation around a constant: ${\theta }_{0}({\rm{\nu }})=\frac{{\gamma }^{2}Hh{\rm{\Delta }}t}{2\pi \nu }(1+{e}^{-T/{\tau }_{0}}\,\cos \,2\pi \nu T)$. The maxima of *θ*_0_(*ν*) appear at *ν* = *m*/*T*, however, they are less pronounced. Accordingly, SW amplitude varies less notably than in the case-I (inset in Fig. 3c). The FWHM of the *θ*_0_(*ν*) peaks can be thus estimated as $\delta \nu ={e}^{T/{\tau }_{0}}/T$.

### Dependence of the SW amplitude on wavevector

The dependence of the SW amplitude on wavevector, Θ_0_(**k**) is governed by the Fourier transform of *h*(**r**) in *k*-space, $\tilde{h}({\bf{k}})$. As the magnetic field of the inverse Faraday effect is proportional to the intensity *I*(**r**) of the circularly polarized beam: *h*(**r**)~*I*(**r**), $\tilde{h}({\bf{k}})\,\,$is determined by *I*(**r**). For uniform illumination of the magnetic film $\tilde{h}({\bf{k}})=\tilde{h}\delta ({\bf{k}})\,\,$and only magnetic oscillations with *k* = 0 are excited. For focused laser beam illumination, SWs with nonzero wavenumbers can be generated. In particular, for a Gaussian beam of radius *r*_0_: $\tilde{h}({\bf{k}})={h}_{0}{r}_{0}\sqrt{\pi }\,\exp (-{{\bf{k}}}^{2}{r}_{0}^{2}/4)$. Actually, the focused laser spot acts here as a kind of SW antenna with wavenumber bandwidth 0 < *k* < *k*_*max*_, constrained by $\tilde{h}({\bf{k}})$:$\,{{\rm{\Theta }}}_{0}({\bf{k}}) \sim \exp (-{{\bf{k}}}^{2}{r}_{0}^{2}/4)$ and, consequently, *k*_*max*_ = 2/*r*_0_. Thus for our experiments with a pump spot radius of *r*_0_ = 4 μm: *k*_*max*_ = 0.5 rad μm^−1^.

If SWs are generated by a sequence of pump pulses, then the cumulative effect modifies Θ_0_(**k**). In this case Θ_0_(**k**) is also governed by *θ*_0_(*v*) and the SW dispersion, *v*(**k**): ${{\rm{\Theta }}}_{0}({\bf{k}}) \sim {\tilde{\theta }}_{0}({\bf{k}})\exp (-{{\bf{k}}}^{2}{r}_{0}^{2}/4)$, where ${\tilde{\theta }}_{0}({\bf{k}})={\theta }_{0}(\nu ({\bf{k}}))$. Therefore, the efficiency of generation of SWs with some particular **k** also depends on the ratio *T*/*τ*.

### Dependence of the spin dynamics on the ratio of *T*/*τ*

**Figure 4:**
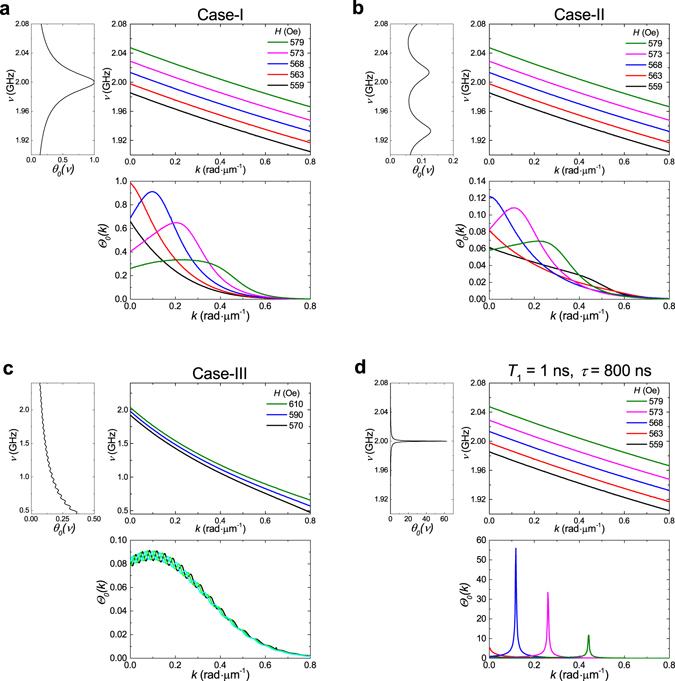
Dependences of the SW amplitude on frequency (upper left panel) and wavenumber (lower panel) for different ratios of the pump-pulse interval and the SW decay time. SWs are generated by the 1GHz-laser in the sample-A (**a**), and by the 80MHz-laser in the sample-A (**b**), and the sample-B (**c**) for different strengths of the external magnetic field. In the upper right panels the SW dispersions are shown. *α*_*SW*_ = 5 × 10^−3^ (**a,b**) and *α*_*SW*_ = 20 × 10^−3^ (**c**), as found from fitting the experimentally measured dependence of the SW amplitude on the external magnetic field. (**d**) Calculations for the magnetic film with ultra low damping, $\frac{T}{\tau }=1.25\times {10}^{-3}$ (*α*_*SW*_ = 1 × 10^−3^).

Let us consider how *θ*_0_(*v*) and Θ_0_(**k**) vary with *T*/*τ* and begin from case-I (*T*_1_ ≪ *τ*_*A*_) when the sample-A is excited by the 1GHz-laser (Fig. 4a). The wavenumber range limited by *k*_*max*_ = 0.5 rad μm^−1^ determines the frequency range of SWs through the dispersion relation *v*(**k**), limiting it to Δ*ν* = 53 MHz width (Fig. 4a, central panel). On the other hand, periodic pumping due to the synchronization phenomenon leads to the sharp peaks in *θ*_0_(*v*) (*δν* = 21 MHz) that are separated by the 1 GHz interval (Fig. 4a, left panel). As Δ*ν* ≪ 1 GHz, only one peak of *θ*_0_(*v*) can appear within the frequency band of SWs. In addition, for optical generation of SWs this frequency band must be tuned such that it contains one of the peaks in *θ*_0_(*v*). This condition is accomplished, for example, for the external magnetic field having a strength from 559 Oe to 578 Oe. This explains why for magnetic fields larger or smaller than this interval, the laser pulses do not excite SWs (see inset in Fig. 3a).

The dependence of the SW amplitude on wavenumber also shows a peak that is related to the peak structure of *θ*_0_(*v*) (Fig. 4a, lower panel). Position and width of this peak depend on the dispersion relation and, therefore, on the external magnetic field. This is a unique feature of the periodic excitation of SWs. Thus, for *H* = 559 Oe and *H* = 563 Oe, the maximum of Θ_0_(*k*_*x*_) is at *k*_*x*_ = 0. At the same time, when *H* = 568 Oe the maximum of Θ_0_(*k*_*x*_) is shifted to *k*_*x*_ = 0.11 rad μm^−1^ and further to *k*_*x*_ = 0.22 rad μm^−1^ and *k*_*x*_ = 0.35 rad μm^−1^ for *H* = 568 Oe and *H* = 579 Oe, respectively. This explains the observed tunability of the SW wavenumber via the external magnetic field since by analyzing the experimentally measured *ξ*(*x*) plots we find just the wavenumbers that correspond to the maxima of Θ_0_(***k***).

If $\frac{T}{\tau }\ll 1$ then the FWHM of Θ_0_(*k*_*x*_) is given by δ*k* ~ 2/(*τv*_*g*_), where *vg* is the SW group velocity. In the case-I, δ*k* ~ 0.2 rad μm^−1^. The narrowest range of generated SW wavenumbers appears for *H* = 563 Oe, where the maximum of Θ_0_(*k*_*x*_) is at *k*_*x*_ = 0: 0 < *k* < 0.11 rad μm^−1^.

When the sample-A is excited by the 80MHz-laser (case-II, *T*_2_ ~ *τ*_*A*_), the function *θ*_0_(*v*) also has relatively sharp peaks (*δν* = 23 MHz) but their separation is much smaller, 80 MHz (Fig. 4b, left panel) and for that reason the SW frequency band always contains one peak of *θ*_0_(*v*) (Fig. 4b, central panel). As a result, SWs are generated for any external magnetic field, even though with varying amplitude (inset in Fig. 3b). The behavior of Θ_0_(*k*) for different magnetic fields is similar to case-I, but δ*k* is a bit larger, and the maximum of Θ_0_(*k*) varies in a slightly smaller range. For example, for *H* = 579 Oe in the case-II, Θ_0_(*k*) does not have any maximum, while in the case-I it has one at *k*_*x*_ = 0.35 rad μm^−1^. Consequently, the synchronization phenomenon for *T*_2_ ~ *τ*_*A*_ still remains prominent.

The case-III corresponding to the sample B excited by the 80MHz-laser (*T*_2_ ≫ *τ*_*B*_), is quite different from the two previous cases. Indeed, SWs are excited with almost constant amplitude that is hardly oscillating around the mean value and *δν* = 405 MHz (Fig. 4c, left panel). This gives an almost monotonic dependence of the SW amplitude on wavenumber, which merely changes for different values of the external magnetic field (Fig. 4c, low panel). Therefore, if the pump pulse impinges on the sample comparatively rarely then one finds a situation similar to the single-pulse regime.

Consequently, the ratio of *T*/*τ* is crucial for the cumulative phenomenon. Thus, for the iron-garnet films with ultra low magnetic losses and small group velocity of SWs the effective damping constant can be as small as *α*_*SW*_ = 1 × 10^−4^ ^30^. In this case periodic pumping would lead to even sharper resonances of Θ_0_(*k*), that allows to excite SWs in narrow band of wavenumbers δ*k* ~ 6 × 10^−3^ rad μm^−1^ and enhancement of the SW amplitude by about 800 times (Fig. 4d).

## Conclusion

We have demonstrated the influence of periodic optical pumping on the spectrum of generated spin waves. The most prominent results are obtained for the case when the interval between laser pulses is much shorter than the decay time of the spin waves. It allows for an efficient synchronization of the magnetic oscillations and, as a result, a significantly modified dependence of the spin wave amplitude on wavenumber. In detail, if a magnetic sample is excited rarely by laser pulses then the maximum of the SW amplitude corresponds to zero wavenumber for any magnetic field. On the contrary, illumination of the sample with frequent pulses modifies the shape of the wavenumber dependence of the SW amplitude: the maximum might be shifted to nonzero wavenumbers, which makes it quite sensitive to the external magnetic field or the repetition rate of the laser pulses. Moreover, the range of wavenumbers of the generated SWs becomes smaller. In addition to that the SW amplitude is significantly enhanced. This precisely adjustable phenomenology is of prime interest for magnonics because it expands considerably the potentialities of ultrafast optical generation and control of SWs.

## Methods

### Calculation of SW excitation by focused laser pulses

The influence of the optical pulses on the magnetization can be described in terms of the amplitude of the effective magnetic field of the inverse Faraday effect, *h*. Due to the Gaussian shape of the laser beam $h({\bf{r}})={h}_{0}\,\exp (-{{\bf{r}}}^{2}/{r}_{0}^{2})$, where *r*_0_ is the radius of the laser beam defined as the distance from the beam axis at which the light intensity is reduced *e* times. Since the probe pulses are incident almost normal to the film, the observed signal (variation of the Faraday angle *Ψ*) is proportional to the angle *θ*_1_ included by the magnetization and the surface of the magnetic film. Therefore, to model the experimental data, the angle *θ*_1_(**r**, *t*) should be calculated as function of **r** and *t*. It can be found as superposition of the SWs generated by *N* pump pulses: ${\theta }_{1}({\bf{r}},t)=\sum _{n=0}^{N-1}{\theta }_{1S}({\bf{r}},t-nT)$, where *θ*_1*S*_(**r**, *t*) corresponds to the SW excited by a single pulse, and *n* is an integer, *n* ≤ *N*. For numerical calculation we used *N* = 100 to achieve a quasi-stationary magnetization dynamics.

The function *θ*_1*S*_(**r**, *t*) is given by integration over wavenumbers **k** = (*k*_*x*_, *k*_*y*_)^16^:

4 $${\theta }_{1}({\bf{r}},t)=\beta \int d{\bf{k}}\,h({\bf{k}})\,\sin ({\bf{k}}{\bf{r}}-\omega ({\bf{k}})t)\,\exp (-\alpha \omega ({\bf{k}})t),$$

where *β* is a proportionality coefficient,$\,h({\bf{k}})={h}_{0}{r}_{0}\sqrt{\pi }\,\exp (-{{\bf{k}}}^{2}{r}_{0}^{2}/4)$ is the Fourier transform of *h*(**r**) in *k*-space, *ω*(**k**) is the SW frequency, and *α* is the Gilbert damping constant. The dispersion of SWs *ω*(**k**) was calculated from the transcendental equations for the backward magnetostatic spin waves^31^.
